# Fusion Imaging of ^18^F-FDG PET and MRI Identified an Inflammatory Esophageal Diverticulum in a Patient with Radioiodine-Refractory Differentiated Thyroid Cancer

**DOI:** 10.3390/diagnostics16020188

**Published:** 2026-01-07

**Authors:** Jiamiao Yang, Peng Zhong, Jiahuan Yang, Xusen Yang, Libo Chen

**Affiliations:** Department of Nuclear Medicine, Shanghai Sixth People’s Hospital Affiliated to Shanghai Jiao Tong University School of Medicine, 600 Yishan Road, Shanghai 200233, China; yangjiamiao0719@sjtu.edu.cn (J.Y.); zhongpeng199441@163.com (P.Z.); yjh6hospital@163.com (J.Y.); yangxusen1998@163.com (X.Y.)

**Keywords:** PET/CT, MRI, fusion image, esophageal diverticulum, radioiodine-refractory differentiated thyroid cancer

## Abstract

A radioiodine-refractory differentiated thyroid cancer patient with rising serum thyroglobulin (Tg) levels underwent ^18^F-FDG PET/CT scan, which showed a hypermetabolic region in the proximal segment of esophagus, leading to ambiguity in diagnosis. MRI was immediately added, and PET/MRI fusion image localized an air-containing lesion interlinked with esophagus with enhanced T2 hyperintense mucosal signal, indicating an inflammatory esophageal diverticulum, which was subsequently verified by endoscopy. This case highlights the added value of PET/MRI image fusion in cases with inconclusive ^18^F-FDG PET/CT findings, requiring no additional tests and utilizing existing software, thereby minimizing the need for invasive procedures.

**Figure 1:**
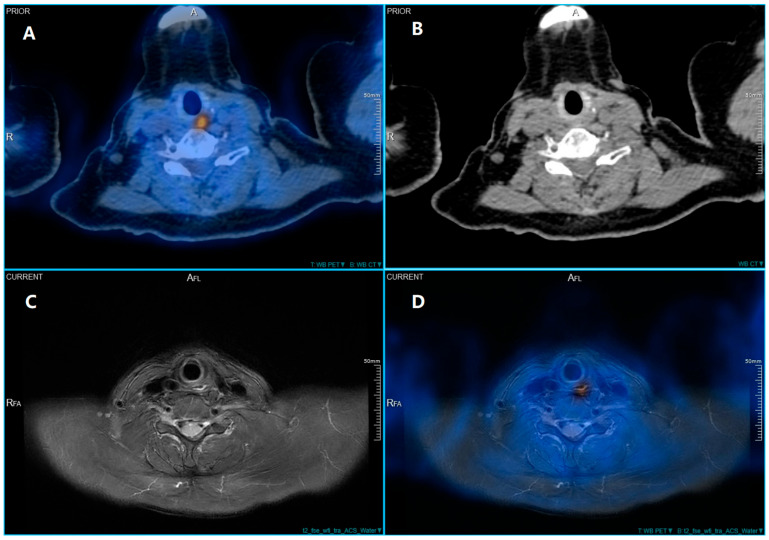
In 2022, a 66-year-old woman with papillary thyroid cancer had a total thyroidectomy and right neck dissection due to nodal metastases, followed by two ^131^I treatments of 150 mCi each. Thyroglobulin levels remained elevated at 41 ng/mL in 2024 and 2025, despite the absence of detectable iodine-avid disease, prompting an ^18^F-FDG PET/CT scan according to ATA guidelines for suspected radioiodine-refractory disease [1]. ^18^F-FDG PET/CT was performed 60 min after intravenous injection of 3.7 MBq/kg of ^18^F-FDG using a Biograph CT scanner (United Imaging, Shanghai, China). CT (120 kV, auto mAs) was used for attenuation correction and anatomical localization. The scan revealed a high-metabolic lesion (SUVmax 7.4) in the proximal segment of esophagus (**A**). However, CT scan failed to identify the lesion (**B**). We further reviewed the patient’s medical history and discovered that in a ^18^F-FDG PET/CT examination conducted a year ago, the patient’s radioactive uptake in this area had already been shown, with SUVmax 7.2 at that time. The lesion was not seen on the CT scan, even though there was noticeable ^18^F-FDG uptake, probably because it was small and lacked significant morphological changes or wall thickening. In cases of inflammatory esophageal diverticula, metabolic activity might be evident without significant structural changes on CT, especially in early or mild inflammation. For improved differentiation of soft tissues, magnetic resonance (MR) imaging was performed on a 3.0T MR with ultra-high magnet homogeneity (United Imaging) 24 h after PET/CT, with axial T2-weighted fat-suppressed sequences (TR/TE = 4430/99.8 ms, slice thickness 5 mm). No contrast agent was administered. In the following MRI, axial T2-weighted imaging with fat suppression showed a left-sided cervical lesion with potential communication into the cervical esophagus. In addition, the left posterior of the esophagus showed high signal, significantly higher than the surrounding normal mucosa, suggesting the possibility of an inflammatory diverticulum (**C**). PET and contrast-enhanced MR T2 axial sequence were further retrospectively fused using uWS-MI and uWS-CT (Version 31.1.1535882, United Imaging). Through software fusion of PET and MR imaging data, the high-metabolic lesion was precisely localized to this area (**D**). Due to the suspected esophageal origin of the hypermetabolic activity, a gastrointestinal endoscopy was subsequently performed. The esophagogastroduodenoscopy confirmed an esophageal diverticulum at the corresponding site (Figure 2). The failure of CT to visualize the diverticulum likely stems from its small size (<1 cm) and the absence of significant wall thickening or mass effect. Inflammatory changes in early-stage diverticulitis are often functional (metabolic) rather than morphologically conspicuous on low-dose, non-contrast CT, which is routinely used for attenuation correction in PET/CT protocols. Alternatively, MRI, particularly T2-weighted sequences with fat suppression, can sensitively reveal mucosal edema and fluid-filled structures. PET/MRI fusion combines the high metabolic sensitivity of ^18^F-FDG-PET with the superior soft-tissue resolution of MRI, making it particularly useful in detecting recurrent or persistent disease in thyroid cancer patients [2]. False-positive ^18^F-FDG uptake in benign inflammatory lesions is a limitation of PET imaging [3]. Esophageal diverticula can harbor food debris and become sites of chronic irritation or infection, leading to mucosal inflammation. Activated inflammatory cells—particularly macrophages and neutrophils—upregulate glucose transporters (e.g., GLUT-1) and exhibit increased glycolytic metabolism, resulting in avid ^18^F-FDG uptake that mimics malignancy [4]. This phenomenon underscores the importance of correlating metabolic findings with high-resolution anatomical imaging to avoid misdiagnosis [5]. For studies aiming to combine functional information from PET with anatomical details from MRI, software fusion of PET and MR imaging data has been proven to be appropriate to enhance the localization and characterization of such lesions [6,7]. As reported before, PET/MRI fusion altered treatment plans in 46% of patients and confirmed plans in 36%, emphasizing its clinical impact in ambiguous cases [8]. The ability of PET/MRI to provide correlative anatomical detail helps differentiate such lesion from thyroid cancer recurrence. PET/MRI fusion can reduce false positives in post-operated thyroid cancer patients with altered anatomy [9]. In this case the hypermetabolic lesion was considered to represent an inflamed esophageal diverticulum. Specifically, this clinical case vividly underscores the incremental diagnostic value of image fusion combining PET and MRI, particularly in scenarios where initial PET/CT evaluations yield inconclusive findings. This software fusion technique facilitates more effective treatment planning and may reduce the need for further imaging or unnecessary procedures.

**Figure 2:**
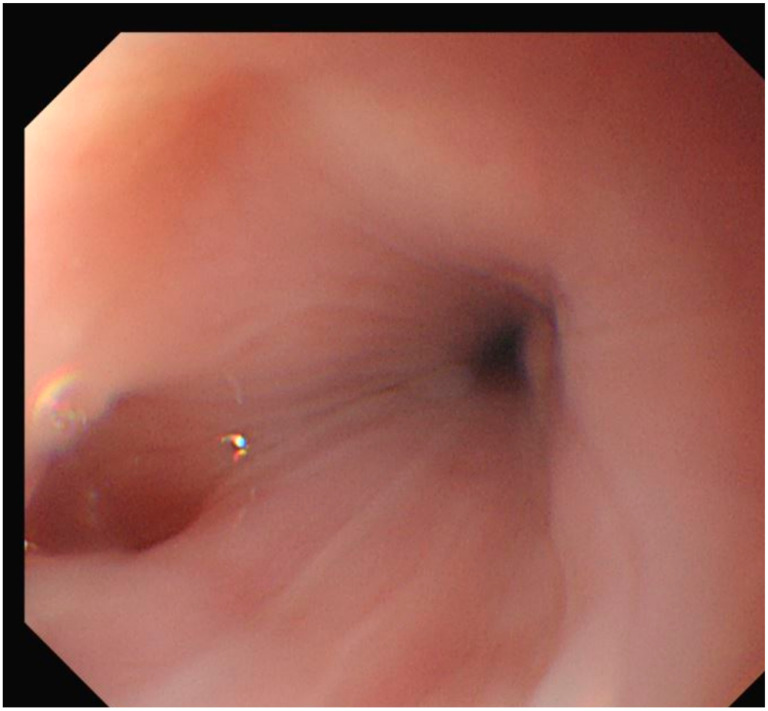
Esophagogastroduodenoscopy revealed diverticulum in the upper esophagus. Although biopsy was not performed due to the classic benign endoscopic and imaging features, the absence of alarm symptoms, stable thyroglobulin levels, and lack of structural progression on follow-up collectively support a non-neoplastic etiology.

## Data Availability

The original contributions presented in this study are included in the article. Further inquiries can be directed to the corresponding author.
